# Rubber-like Quasi-thermosetting Polyetheramine-cured Epoxy Asphalt Composites Capable of Being Opened to Traffic Immediately

**DOI:** 10.1038/srep18882

**Published:** 2016-01-06

**Authors:** Yang Kang, Qiang Wu, Rui Jin, Pengfei Yu, Jixiang Cheng

**Affiliations:** 1 College of Water Resources and Architectural Engineering, Northwest A&F University, Yangling, Shaanxi, 712100, P R China; 2 Jinan Urban Construction Group, Shandong, 250031, P R China

## Abstract

This paper reports the facile preparation, mechanical performance and linear viscoelasticity of polyetheramine-cured rubber-like epoxy asphalt composites (EACs) with different asphalt contents. Compared with previous EACs prepared via complex chemical reactions and time-consuming high-temperature curing, the EACs reported here were obtained by using a compatible, bi-functional polyetheramine and a simple physical co-blend process, which make the EACs feasibly scalable for production at a lower cost. The EACs were cured for 1 h at 160 °C and 3 d at 60 °C; therefore, these composites can be opened to traffic immediately. The EACs have a much greater temperature stability than common thermoplastic polymer-modified asphalt composites from −30 °C to 120 °C, but their complex shear moduli at higher temperatures slightly decrease instead of remaining constant when temperatures are greater than 80 °C, especially for the higher asphalt content composites; that is, these composites are quasi-thermosetting. Wicket plots illustrate that the EACs reported here are thermorheological simple materials, and the master curves are constructed and well-fitted by generalized logistic sigmoidal model functions. This research provides a facile, low-cost method for the preparation of polyetheramine-cured EACs that can be opened to traffic immediately, and the concept of quasi-thermosetting may facilitate the development of cheaper EACs for advanced applications.

Asphalt has been used to pave roads for hundreds of years, owing to its adhesive and waterproof properties and its ability to be produced in large quanities^1^. As a typical viscoelastic material, asphalt flows at higher temperatures and becomes brittle at lower temperatures. To reduce its thermal susceptibility, physical co-blends and chemical modifications have been employed. Physical modifiers include styrene–butadiene–styrene (SBS), polypropylene, polyethylene, nanomaterials, and fibers. Sulfur, maleic anhydride and dicarboxylic acids, polyphosphoric acid, epoxy resin, thiourea and other functionalized SB/SBS polymers have been reacted with the active ingredient of asphalt to chemically improve the paving performance of asphalt^2^,^3^,^4^,^5^,^6^,^7^,^8^. Although these methods have improved the paving performance of asphalt to some extent, they cannot currently meet the rigorous demands of superior traffic volumes because of their thermoplastic nature; therefore, thermosetting epoxy asphalt composites (EACs) have been thought to be a better choice to improve the durability of busy roads^9^,^10^,^11^.

Typically, EACs are two-component systems that result from the reaction of asphalt with curing agents (component **A**) with epoxy resins (component **B**). According to their curing agents, EACs are categorized as amine systems or acid systems^9^,^12^,^13^,^14^,^15^. The typical laboratory curing conditions of an acid EAC system are 4 h at 120 °C; however, at the paving site where the EAC is exposed to the atmosphere, temperatures cannot be maintained at 120 °C for more than 1 h during the paving process; consequently, to achieve a performance that is equivalent to that in the laboratory, according to the empirical Arrhenius equation of chemical reaction rate theory, sites paved via the acid EAC systems should be naturally maintained in high summer for about 45 days^16^,^17^,^18^,^19^,^20^,^21^. A typical construction process is shown in Fig. 1. Moreover, both amine and acid curing agent systems are prepared through complex synthesis routes or use unavailable raw materials, which greatly increases costs^12^,^22^,^23^,^24^. Yin *et al.* have reported EACs cured by aliphatic amine (octadecylamine, CH_3_(CH_2_)_16_CH_2_NH_2_) for 1 h at 150 °C and 3d at 60 °C^25^. Undoubtedly, this is a promising approach for the development of EACs capable of being opened to traffic immediately. Still, the poor compatibility among octadecylamine, asphalt and the epoxy resin restricts the asphalt content to 40.0 g per 100.0 g of epoxy resin, which results in a higher cost.

To overcome these drawbacks, a polyetheramine with longer chains and polar ether groups was introduced to prepare EACs by physical co-blending with the asphalt. Benefiting from the compatible and bi-functional curing agent, the simple physical co-blending process and the affordability of the specific polyetheramine raw materials, the EACs reported here are cheaper and easier to further optimize for large-scale production for use in expressways. Furthermore, the curing conditions of 1 h at 160 °C and 3d at 60 °C allow the road to be immediately opened to traffic.

## Results and Discussion

**Figure 2:**
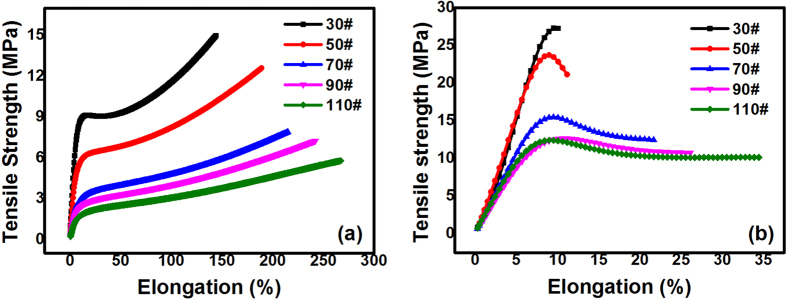
Effect of asphalt content on the tensile properties of polyamine cured EACs. (a) Tensile strength vs. rupture elongation at 20 °C; all EACs have rubber-like elastic characteristics; (**b**) Tensile strength vs. rupture elongation at 0 °C; all EACs are flexible at 0 °C.

Conventionally, the characterization method of EACs has been borrowed from the characterization of plastics, for example, ASTM D 638–2010: Standard Test Method for the Tensile Properties of Plastics^12^,^13^,^16^,^17^,^18^,^19^,^20^,^21^,^25^. To compare the performance of polyetheramine-cured EACs, direct tensile tests were performed with a universal tester at 20 °C and 0 °C, as shown in Fig. 2 (a is at 20 °C, and b is at 0 °C). All samples had elastic properties similar to those of anhydride cured thermosetting EACs at 20 °C; as the asphalt content increases, the tensile strength decreases and the rupture elongation is enhanced^16^. Previous studies have demonstrated that the addition of asphalt has no influence on the extent of the epoxy curing reaction under the same curing conditions^25^. Therefore, changes in the mechanical properties were ascribed to the decrease in the relative volume ratio of the epoxy-polyetheramine crosslinked chemical networks in the cured EACs when the filled asphalt content increased. Explicitly, a greater relative volume ratio of the filled asphalt EACs resulted in the EACs behaving more like viscoelastic asphalt. Additionally, the absolute values of the rupture elongation at 20 °C were equivalent to measurements from previous studies, and the tensile strength values were greater than the values from previous studies^24^,^25^. Specifically, the EACs reported here exhibited flexible characteristics at a temperature of 0 °C, as shown in Fig. 2b.

To understand the paved performance of polyetheramine-cured EACs, their rheological properties should be carefully studied^26^,^27^. The rheological properties of asphalt composites are typically characterized by static experiments, such as relaxation and creep tests, and dynamic experiments, such as temperature sweeps under fixed frequency and frequency sweeps under different temperatures. Moreover, the static and dynamic rheological data can be mutually converted in the linear viscoelastic (LVE) region^28^. Temperature sweeps are used to determine the temperature susceptibility of polyetheramine-cured EACs with asphalt content increasing in the LVE region. Additionally, experimental rheological data within a proper frequency loading range obtained from a series of temperatures can be shifted relative to the reduced frequencies to build a master curve. From the master curve, it is possible to interpolate the modulus over an expanded frequency range^29^. Master curves can be built by the so-called time-temperature superposition principle (TTSP) when the frequency sweep tests are performed in the LVE region and the materials are thermorheologically simple^26^,^27^,^28^. Therefore, to obtain a universal master curve, it is very important to ensure that the frequency sweeps are carried out in the LVE region and that the polyetheramine-cured EACs are thermorheological simple materials.

**Figure 3:**
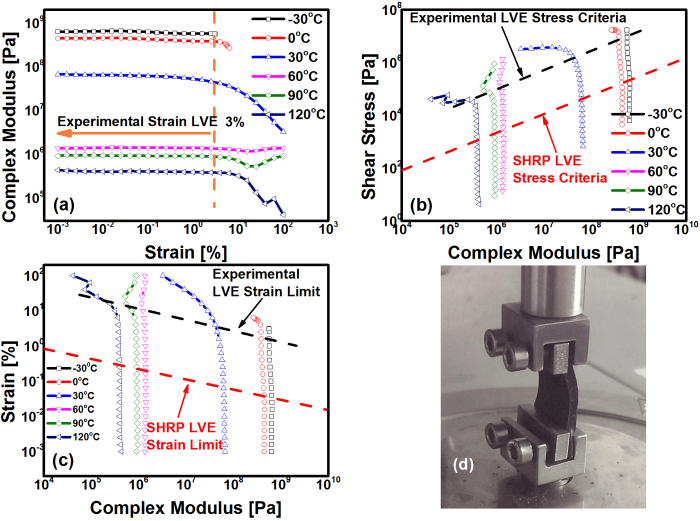
Linear viscoelastic (LVE) regions of polyetheramine-cured EACs at different temperatures determined by strain sweep experiments (take 30# for example). (**a**) Complex modulus vs. strain curves; (**b**) Stress vs. complex modulus curves; (**c**) Complex modulus vs. strain curves; (**d**) Customized SRF5 solid torsion geometry used during all experiments.

Figure 3a shows the strain sweep results of 30# (30.0 g of asphalt per 100.0 g of polyetheramine, see Methods); when the strains were less than 3% (Fig. 3a), the sample behaved in the LVE region. In the low-temperature range below 0 °C, which is limited by the equipment, the shear strain was no more than 3%. It is noteworthy that the sample remained in the LVE region when the shear stress was near 4 × 10^4^ Pa at 120 °C (Fig. 3b), which means that the LVE properties at 120 °C are equivalent to the properties of multigrade 30/50 bitumen at 30 °C^30^. Moreover, all EACs with different contents of asphalt had similar LVE characteristics. Therefore, we selected a strain of 0.3% as the frequency sweep parameter.

In fact, the experimental LVE results were consistent with the SHRP (strategic highway research program) stress and strain LVE limits, as shown in Fig. 3b and Fig. 3c. These results are the criteria for penetration grade bitumen materials and are functions of the complex modulus, as defined by the following equations:


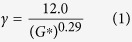



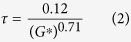


where τ is the shear stress in Pa and is measured by a dynamic shear rheometer (DSR), γ is the shear strain in % and is measured by the DSR, and G^*^ is the complex modulus in Pa^30^. Clearly, the SHRP LVE limits are relatively conservative compared with the experimental results, as shown in Fig. 3b,c. Additionally, Fig. 3d shows the customized SRF5 solid torsion geometry measured by an Anton Paar MCR302.

**Figure 4:**
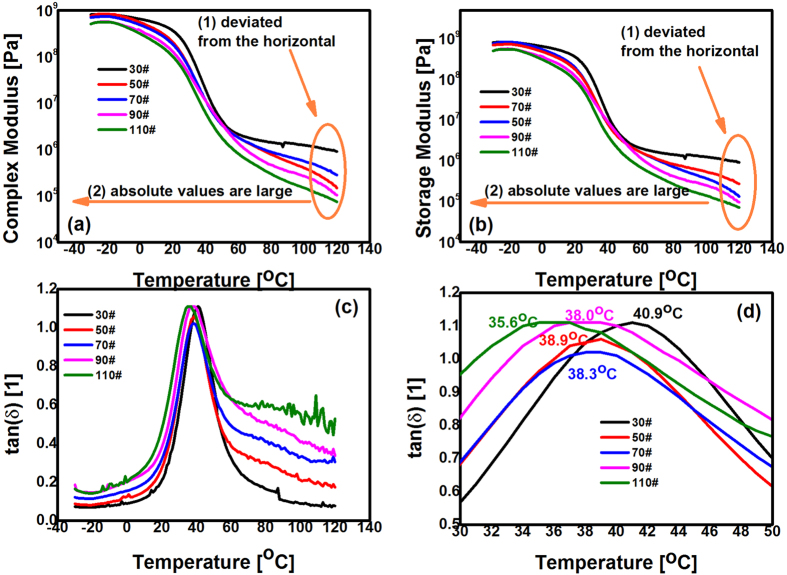
Effect of the asphalt content on the temperature stability of the polyetheramine-cured EACs. (**a**) Complex moduli vs. temperature curves (note the two characteristics of quasi-thermoset materials); (**b**) Storage moduli vs. temperature curves; (**c**) tan(δ) vs. temperature curves; (**d**) tan (δ) vs. temperature curves from 30 °C to 50 °C.

As presented in Fig. 4a,b, with an increase in the asphalt content, over the whole range of experimental temperatures, the absolute values of the complex modulus (G^*^) and the storage modulus (G’) increased. Similarly to the tensile tests (Fig. 2), the reason for this is attributed to the decreasing relative volume ratio of the epoxy asphalt chemically crosslinked networks while the asphalt content increases. Clearly, when EACs had lower relative volume ratios of their crosslinked networks, the EACs behaved more like asphalt. However, it was noticeable that, in the range of higher temperatures (>80 °C), the complex shear modulus (G^*^) and storage modulus (G’) simultaneously decreased slightly to 1 × 10^5^ Pa. These results are quite different from the characteristics of anhydride cured thermosetting EACs, in which G* and G’ both remain constant at approximately 5 × 10^5^ Pa in the higher temperature range greater than 80 °C. These findings are also quite different from the characteristics of the traditional thermoplastic modified asphalt composite, in which G^*^ and G’ decrease quickly to less than 5 × 10^2^ Pa in this temperature range^31^. Although G^*^ and G’ of polyetheramine-cured EACs decreased with temperature, their absolute values were still much greater than those of thermoplastic modified asphalt composites and were still within the same order of magnitude as those of the thermosetting EACs^31^. Therefore, we designate these polyetheramine-cured EACs as being quasi-thermosetting.

As Fig. 4c illustrates, in temperature ranges below 30 °C and over 50 °C, the absolute value of the damping factor (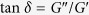) increased with the asphalt content because the components were proportional to the volume ratio of the filled viscous asphalt. Notably, in the temperature range from 30 °C to 50 °C, the peak values of the damping factors first became smaller and then increased as the asphalt content increased, as shown in Fig. 4d. This result is because of the asphalt being added to the EACs at a lower content level, which is akin to a group of hard spheres being added to the chemically crosslinked polyetheramine-cured EACs. Subsequently, the storage modulus increased to a value greater than the loss modulus, thus resulting in an increase in the value of the damping factor. With an increase in the asphalt content, the inner interactions of the asphalt spheres were enhanced, and the viscous character of asphalt became more apparent; that is, the value of the apparent damping factor increased. Furthermore, Fig. 4d shows that all the damping factors peaks for all the EACs were located at a similar temperature of approximately 38 ± 2 °C. Additionally, the peak locations of the damping factors (i.e., glass transition temperatures, T_g_) were related to the asphalt content. We believe that it is the plasticization effect of the added asphalt that makes the corresponding glass transition temperatures decrease with increasing asphalt content.

Specifically, there was no evident phase separation because the peaks of the damping factors were still sharp, with asphalt content up to that of sample 110# (Fig. 4c). This result is attributed to the longer molecule chains and the polarity of the ether groups (-O-) of the selected polyetheramine that increase the compatibility of the asphalt, the amine curing agent and the epoxy resin. As a result, the polyetheramine-cured EACs reported here can accommodate more asphalt than the previous EACs and thus lower the EAC cost^25^.

**Figure 5:**
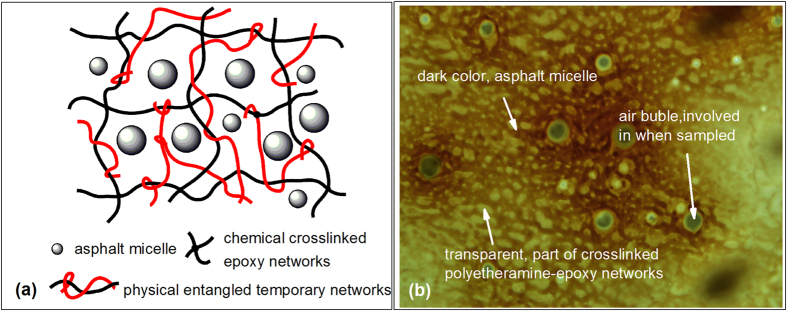
Microscopic structure of the polyetheramine-cured quasi-thermosetting EACs. (**a**) Presumed microstructure; (drawn by the corresponding author); (**b**) Microscope image of 30# polyetheramine-cured EACs observed with an Olympus BX51.

From the viewpoint of the chemical reactions, bi-functional polyetheramine was polycondensed with bi-functional epoxy resin E-51, which was accelerated by DMP-30, and with time, a three-dimensional chemically crosslinked network filled asphalt was formed. Therefore, we believe that quasi-thermosetting characteristics can be attributed to the light or sparse chemically crosslinked epoxy-polyetheramine networks that were formed. Specifically, we hypothesize that the microscopic structure of the quasi-thermosetting EAC should be a sparse chemically crosslinked epoxy-polyetheramine network filled or hung with physically entangled long un-crosslinked epoxy-polyetheramine chains and asphalt micelle, as shown in Fig. 5a. The microscopic structure observed with an Olympus BX51 verified our hypothesis (Fig. 5b). Notably, the networks of quasi-thermosetting EACs reported in this work differ from the bimodal networks of thermosetting EACs in previous studies^16^,^31^. Nevertheless, the quasi-thermosetting EACs would maintain their strength before decomposing when the temperature is increased; that is, their chemically crosslinked networks would provide an eternal storage modulus much greater than 0 Pa. This critical characteristic of quasi-thermosetting EACs is similar to that of thermosetting EACs but is different from that of traditional thermoplastic EACs^16^,^31^.

**Figure 6:**
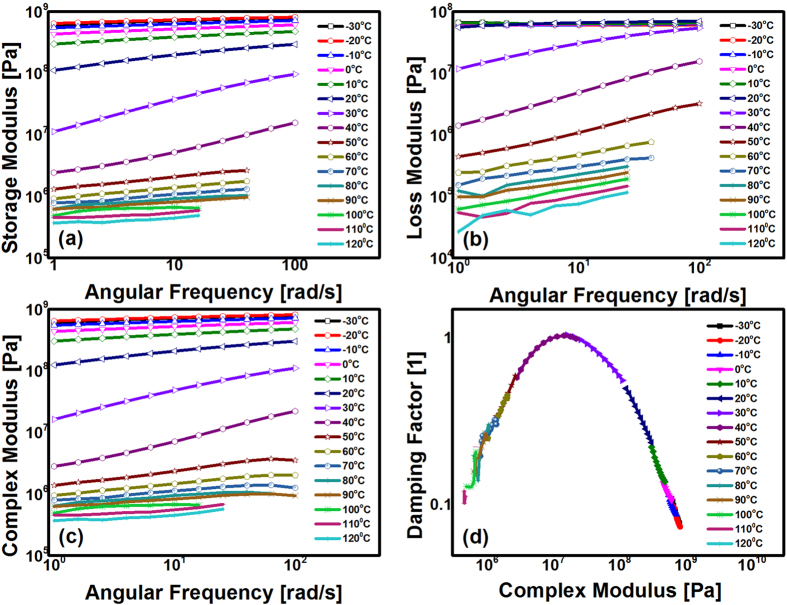
Frequency sweep results at a series temperature from −30 °C to 120 °C measured every 10 °C (take 30# as an example). (**a**) Storage modulus vs. frequency; (**b**) Loss modulus vs. frequency; (**c**) Complex modulus vs. frequency; (**d**) Wicket plot (log-log plot of tan (δ) vs. complex modulus).

As presented in Fig. 6a–c, the frequency sweep results indicated that sample 30# had a greater frequency sensitivity at temperatures from 20 °C to 40 °C, which implies that there is a relaxation process in this temperature range, as shown in Figs 3c and 4d^ ^28. The frequency sweep results for all the EACs were similar to those of sample 30#.

A wicket plot, which is a log-log plot of the damping factor vs. the complex modulus, was used to confirm whether the TTSP can be used to construct master curves. If the wicket plot is a continuous curve (e.g., inverted U-shape, as shown in Fig. 6d), the TTSP is tenable; if the wicket plot has bifurcations, the TTSP cannot be used to superpose master curves because the corresponding material is inhomogeneous^32^,^33^. In essence, an inverted U-shape wicket plot indicates that the material has a unique relaxation mechanism in the studied system. Figure 6d demonstrates that the TTSP was reliable for building a master curve with material 30#, and the other EACs had a similar inverted U-shape wicket plot. Therefore, we constructed their corresponding master curves by using the TTSP.

**Figure 7:**
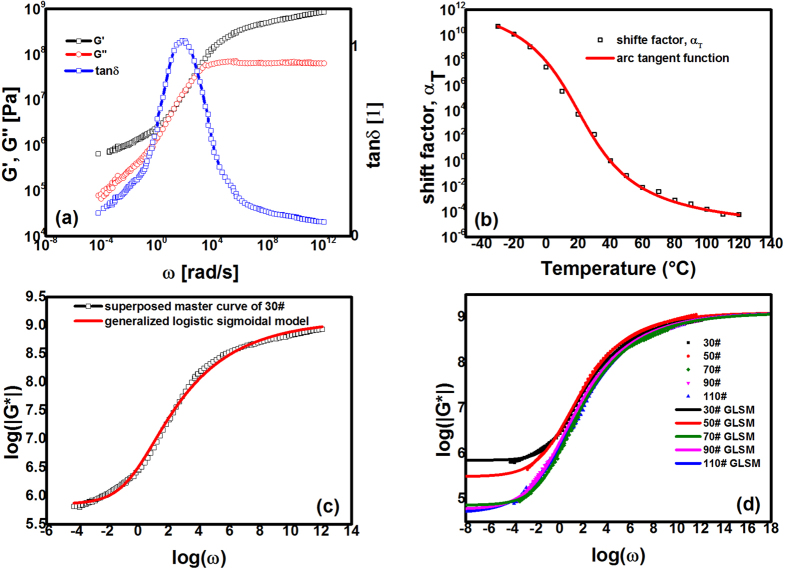
(**a**) Master curve built by using the TTSP (take 30# as an example, T_ref_ = 40 °C); (**b**) Shift factor for the master curve for 30#, and the fitting was performed by using the arc tangent function. The arc tangent function fits formally well; (**c**) Master curve of log (|G^*^|) vs. log (ω) fitted by using the GLSM (take 30# as an example, T_ref_ = 40 °C); (**d**) Experimental data with GLSM simulated function curves.

For example, Fig. 7a shows the successful construction of a master curve from the frequency sweep results by using the TTSP. As shown in Fig. 7a, the polyetheramine-cured EAC exhibited elasticity throughout the entire range of the frequency at the reference temperature of 40 °C (G’ ≥ G’’ during the whole reduced frequency range). When ω → 0, the storage modulus remained greater than 1 × 10^5^ Pa. This result implies that the quasi-thermosetting EAC has superior low-frequency performance compared with traditional thermoplastic modified asphalt composites^34^. In addition, the entire plot appeared similar to a mirror image of its temperature sweep result, as shown in Fig. 4b,c. This phenomenon can be seen as the embodiment of the TTSP. All the EACs had similar master curves, as constructed by using the TTSP.

The corresponding shift factors are plotted in Fig. 7b. Clearly, the WLF and Arrhenius equations, which both have hyperbolic forms, do not fit the shift factor α_T_ well. Another Kaelble modified WLF equation also fails to fit the shift factors because the Kaelble-modified WLF equation is not good at fitting non-symmetrical data^35^. Fortunately, a so-called arc tangent fit function has been presented to simulate such inverted S-shape data^36^:


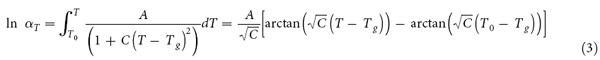


where T_0_ is the reference temperature, T_g_ is the glass transition temperature, and A and C are parameters. As shown in Fig. 7b, the arc tangent function adequately fits the shift factor, and the other sample EAC fitting results are listed in the Table 1. However, the fitted parameters of T_g_ evidently differ from the experimental results, as shown in Fig. 4a–c (approximately 38 ± 2 °C); thus, the arc tangent function fits formally well.

The mathematical model fitted to the master curve is important for the development of pavement design. In materials research, these models are called constitutive equations. Many mathematical models have been built to properly describe types of master curves for bituminous materials, such as binders and concretes^26^,^29^. These models can be classified into two groups: empirical algebraic equations and mechanical element models (or analogical models). There is no essential distinction between these empirical models. These models were created solely for fitting the different shapes of rheological master curves that are determined by the nature of the asphalt materials considered. The generalized Maxwell, generalized Kelvin, Huet, Huet–Sayegh and 2S2P1D are the main mechanical element models^26^. The most commonly used empirical algebraic models are the CA, CAM, modified CAM, and standard Sigmoidal models, all of which have been adopted in the MEPDG (Mechanistic Empirical Pavement Design Guide, AASHTO)^26^.

For convenience of industrial applications, Rowe *et al.* have proposed a generalized logistic sigmoidal model (GLSM) to predict the TTSP-constructed master curves of the complex moduli of asphalts:


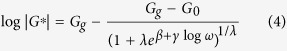


where G_g_ is the limiting maximum value of |G^*^| (maximum asymptote value), G_0_ is the limiting minimum value of |G^*^| (minimum asymptote value), β and γ define the shape between the asymptotes, and the location of the inflection point (when λ = 1, the point satisfied with β + γ log ω = 0), and λ allows the shape to be non-symmetrical (based on log ω = 0)^26^,^37^. This model predicted quasi-thermosetting EAC 30# well over all frequency ranges, as shown in Fig. 7c. All the EACs were well fitted by using the GLSM, and their fitted parameters are listed in Table 2. (The parameters of G_g_ were arbitrarily set to be a constant at 9.1, according to the temperature sweep results shown in Fig. 4a.) It is noticeable that GLSM fit the lower frequency data well. Table 2 shows that G_0_ apparently decreased with an increase in the asphalt content, and this tendency was also observed in the temperature sweep results, as shown in Fig. 4a,b. The simulated curves are presented in Fig. 7d, together with the experimental data from all the EACs.

## Conclusions

The preparation of mediate-temperature post-cured EACs with a simple physical co-blend of asphalt and a bi-functional polyetheramine curing agent was presented and compared with results from previous studies. The longer molecule chains and the polarity of the ether groups (-O-) in the selected polyetheramine increased the compatibility of the asphalt, the amine curing agent and the epoxy resin. As a result, the polyetheramine-cured EACs presented here can accommodate more asphalt than previous EACs, which therefore lowers the cost of the EACs. Furthermore, the polyetheramine-cured EACs can be cured under the conditions of 1 h at 160 °C and 3d at 60 °C, which means that the EACs reported here can be opened to traffic immediately after paving. Direct tensile tests showed that the tensile strength decreases and the rupture elongation increases with asphalt content, as observed for anhydride cured thermosetting EACs. The strain sweep results illustrated that the LVE regions are small (less than 3%) in the experimental temperature range from −30 °C to 120 °C. However, the material’s linear shear stress limits at 120 °C were equivalent to those of multigrade 30/50 bitumen composites at 30 °C, which implies they also have extraordinary high temperature performance. Temperature sweep results indicated that the homogeneous EACs have a much greater temperature stability than traditional thermoplastic polymer modified asphalt composites from −30 °C to 120 °C, but their terminal moduli slightly decreased instead of remaining constant when the temperature increased to greater than 80 °C. This result was especially noticeable in the higher asphalt content EACs; thus, the EACs are quasi thermosetting. All these characteristics are attributed to the light chemically crosslinked epoxy-polyetheramine networks filled with or hung by quantities of physically entangled long un-crosslinked epoxy-polyetheramine chains and asphalt micelles.

The wicket plots showed that the quasi-thermosetting polyetheramine-cured EACs are thermorheological simple materials. Therefore, master curves were superposed by using the TTSP. Furthermore, their constitutive equations were built by using the empirical GLSM functions for the convenience of engineering applications.

## Methods

### Materials

JEFFAMINE^®^ M-1000 (CH_3_(OCH_2_CH_2_)_19_(OCH_2_CHCH_3_)_3_NH_2_, Huntsman, USA), DMP-30 (CAS No. 90–72–2; 2,4,6-tri(dimethylaminomethyl)phenol, Shanfeng Chemicals, Changzhou, China), 90# asphalt (Dushanzi, China) and epoxy resin E-51 (diglycidyl ether of bisphenol A, Wuxi Resins, Wuxi, China) were used as received.

### Preparation of polyetheramine-cured epoxy asphalt composites

Reactions were carried out in a wide-mouthed glass flask fitted with a mechanical stirrer and a thermocouple. First, the asphalt sample (30.0 g, 50.0 g, 70.0 g, 90.0 g, and 110.0 g, respectively) was added to the flask loaded with 100.0 g 160 °C JEFFAMINE^®^ M-1000. The mixture was stirred for 10 minutes at 160 °C to achieve homogeneity. Then, 1.20 g of DMP-30 was dropped into the flask; and the resulting mixture was agitated for 10 minutes. The end product was designated as component A, and the epoxy resin E-51 was designated component B.

Prepared component A and 70.0 g of component B were heated to 160 °C, were mixed and were sheared for 1 min. Then, the mixture was poured into a heated (160 °C) steel mold and baked (160 °C) in an oven to cure for 1 h. The mold was placed in another 60 °C oven for 3 days. The cured amine cured EAC was demolded, and the final products were labeled 30#, 50#, 70#, 90# and 110#.

### Characterization of polyetheramine-cured epoxy asphalt composites

Cured EACs were observed with a BX51 (Olympus, Japan). Samples were prepared according to the following procedure: (1) a drop of the resultant of sheared A and B mixture was obtained with a capillary tube; (2) a drop of the mixture was placed on the surface of a hot object glass slide; and (3) the drop was coated with another glass slide and placed in a 160 °C oven for 1 h and then placed in another 60 °C oven for 3 days.

Direct tensile tests were carried out using a WDW-2000 universal tester (Changchun Kexin Test Machine, China), according to ASTM D638-2010, at the specified temperature. Six different specimens were tested for each sample.

Strain sweep tests were conducted by using a MCR302 dynamic shear rheometer (Anton Paar, Austria) using a customized SRF5 solid torsion geometry (Fig. 3d) at an oscillation frequency of 10 rad/s and strains increasing from 0.001% to 30%. The sample dimensions were 10 mm ×10 mm ×2 mm.

Temperature sweep experiments were conducted by using the MCR302 with the customized SRF5 solid torsion geometry. The selected strain was 0.3%; the oscillation frequency was 10 Hz; the temperature range was from 120 °C to −30 °C. The measurements were conducted for 10 minutes to eliminate thermal and stress histories, and the sample dimensions were 10 mm ×10 mm ×2 mm.

Frequency sweep experiments were conducted with the MCR302 with the customized SRF5 solid torsion geometry. The selected strain was 0.3%, and the frequency increased from 1 rad/s to 100 rad/s; the temperature decreased from 120 °C to −30 °C using a 10 °C step. The temperatures were held stable for 10 minutes per step, and the sample dimensions were 10 mm ×10 mm ×2 mm. Each rheological experiment above mentioned was conducted with three replicates.

## Additional Information

**How to cite this article**: Kang, Y. *et al.* Rubber-like Quasi-thermosetting Polyetheramine-cured Epoxy Asphalt Composites Capable of Being Opened to Traffic Immediately. *Sci. Rep.* **6**, 18882; doi: 10.1038/srep18882 (2016).

## Figures and Tables

**Figure 1:**
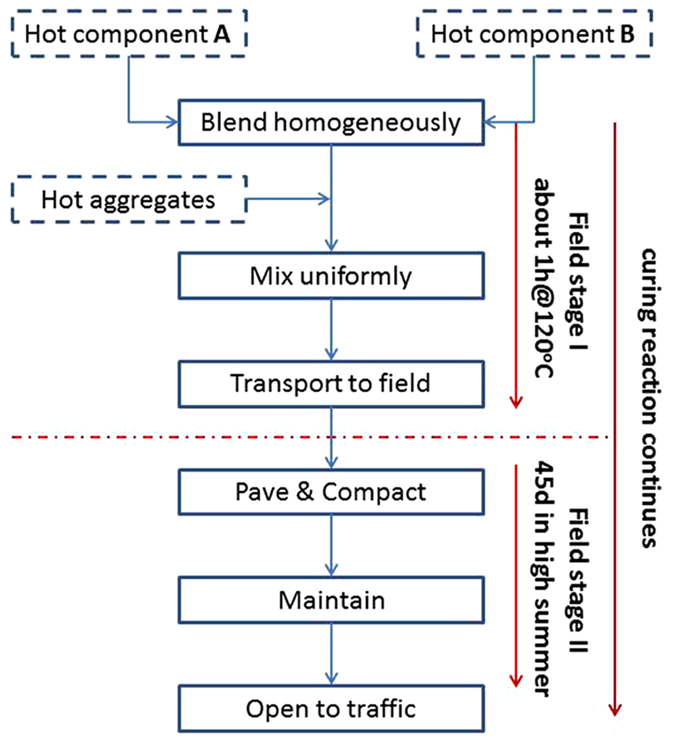
EAC construction process. EACs are preserved in blending equipment and trucks for approximately 1 h at 120 °C (Field stage I). When paved, typically, the asphalt is maintained in high summer for about 45 days (Field stage II). While in the lab, to simulate the two field stages, the blended binder is maintained at 120 °C for 4 hours.

**Table 1:** Parameters fitted by arc tangent functions of shift factors.

No.	A	C	T_0_	T_g_	*R*^2^
30#	−0.5267	1.31E-3	40	20	0.9999
50#	−0.5940	2.70E-3	40	20	0.9986
70#	−0.4829	1.06E-3	40	15	0.9969
90#	−0.4574	1.06E-3	40	15	0.9999
110#	−0.6643	2.57E-3	40	18	0.9982

**Table 2:** Parameters fitted by the generalized logistic sigmoidal model (GLSM).

No.	G_0_	G_g_	β	γ	λ	*R*^2^
30#	5.855	9.1	−1.066	0.994	3.734	0.9981
50#	5.493	9.1	−0.699	0.729	2.338	0.9995
70#	4.866	9.1	−0.531	0.823	3.183	0.9989
90#	4.777	9.1	−0.297	0.681	2.572	0.9984
110#	4.695	9.1	−0.501	0.550	1.947	0.9984
